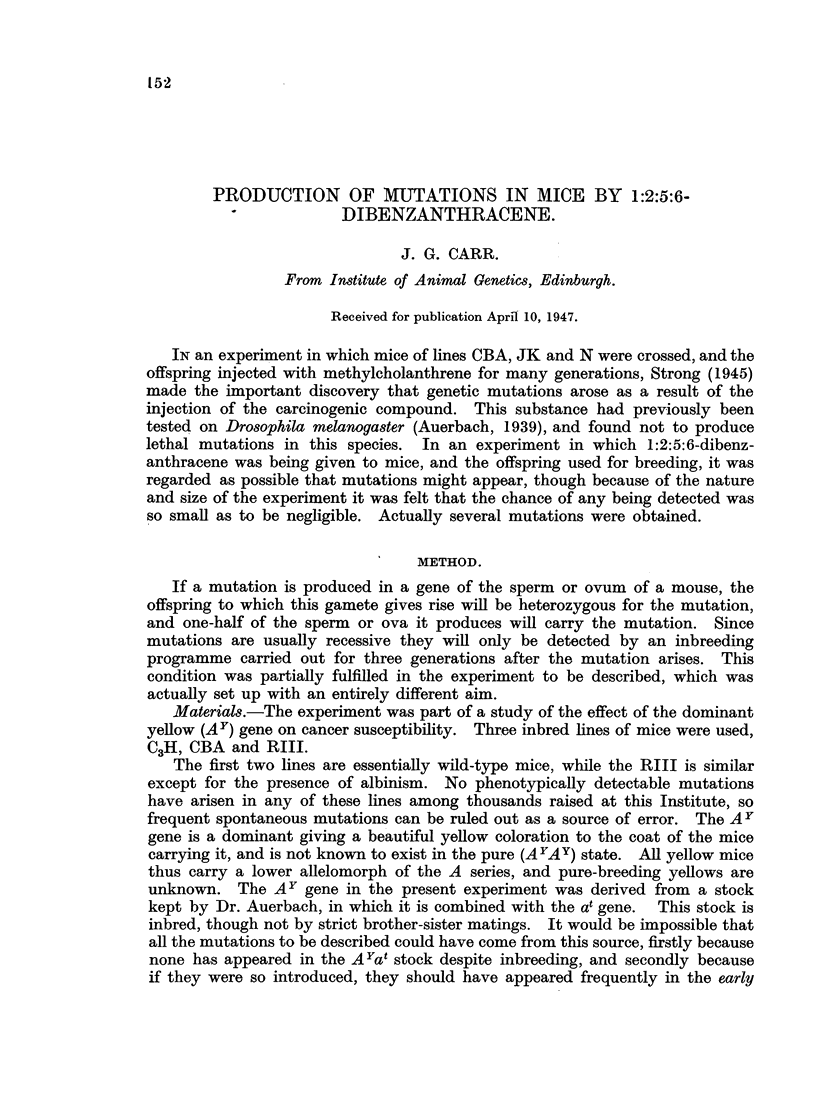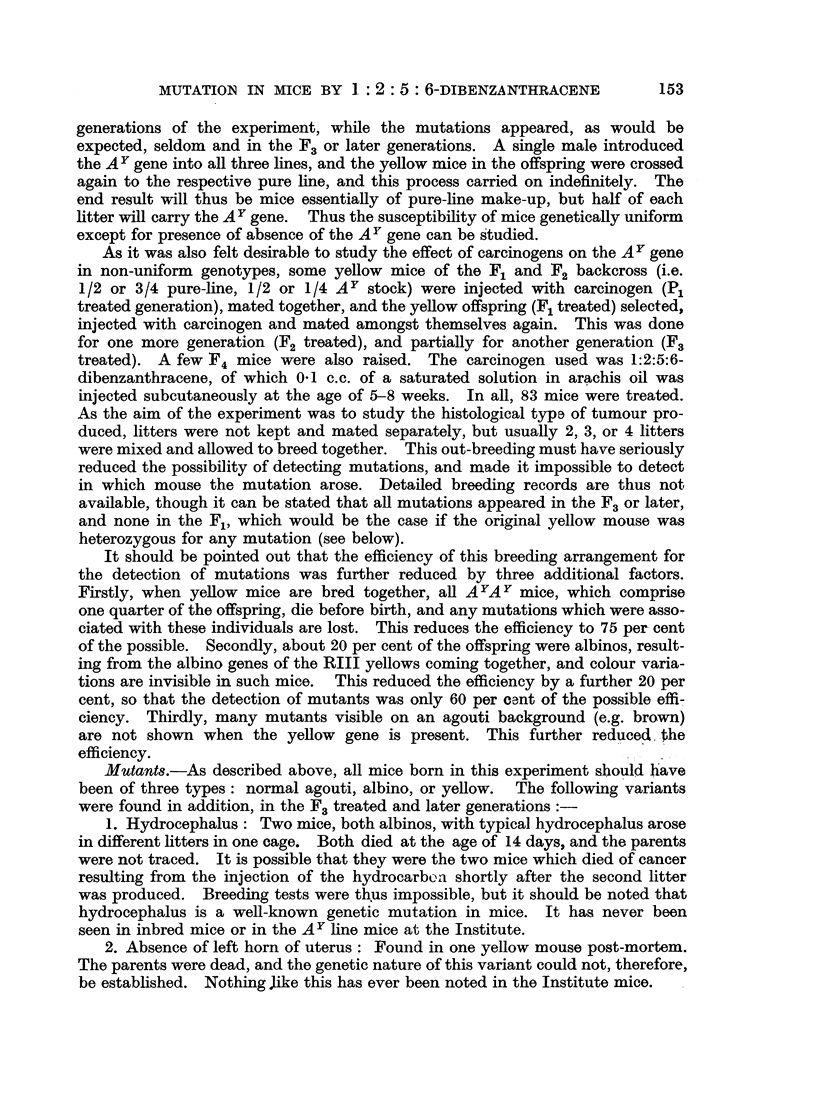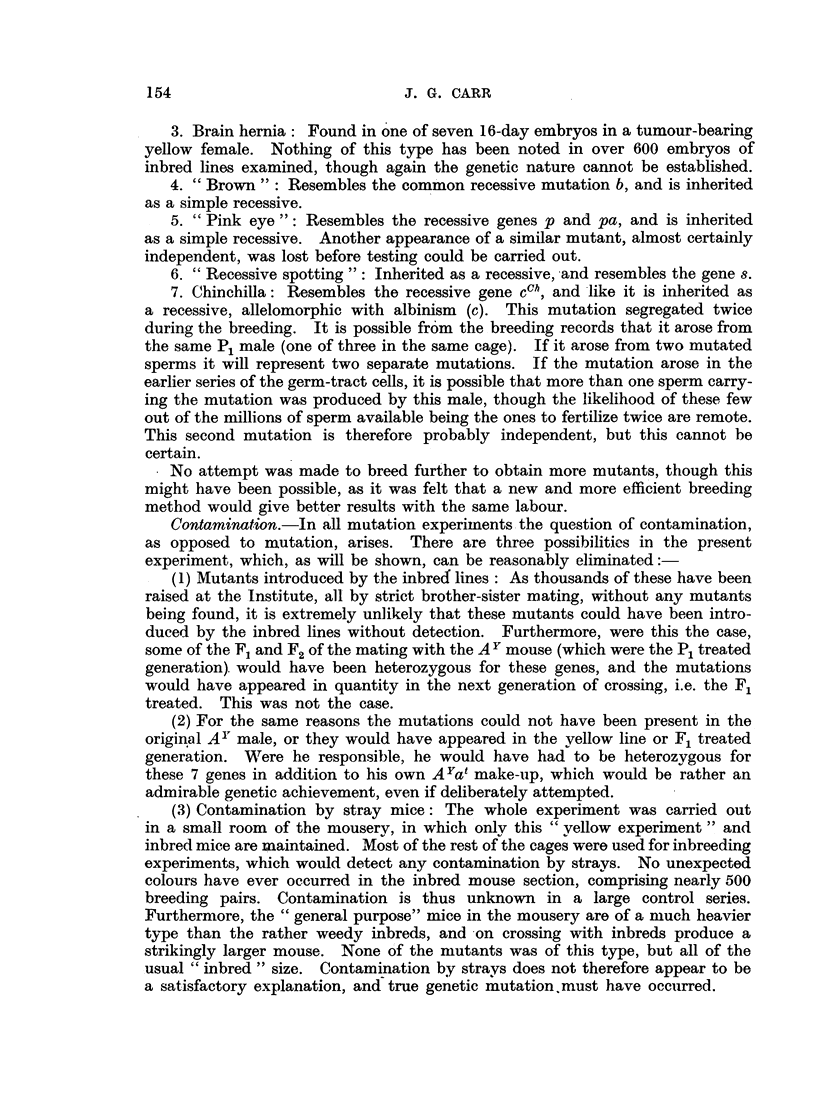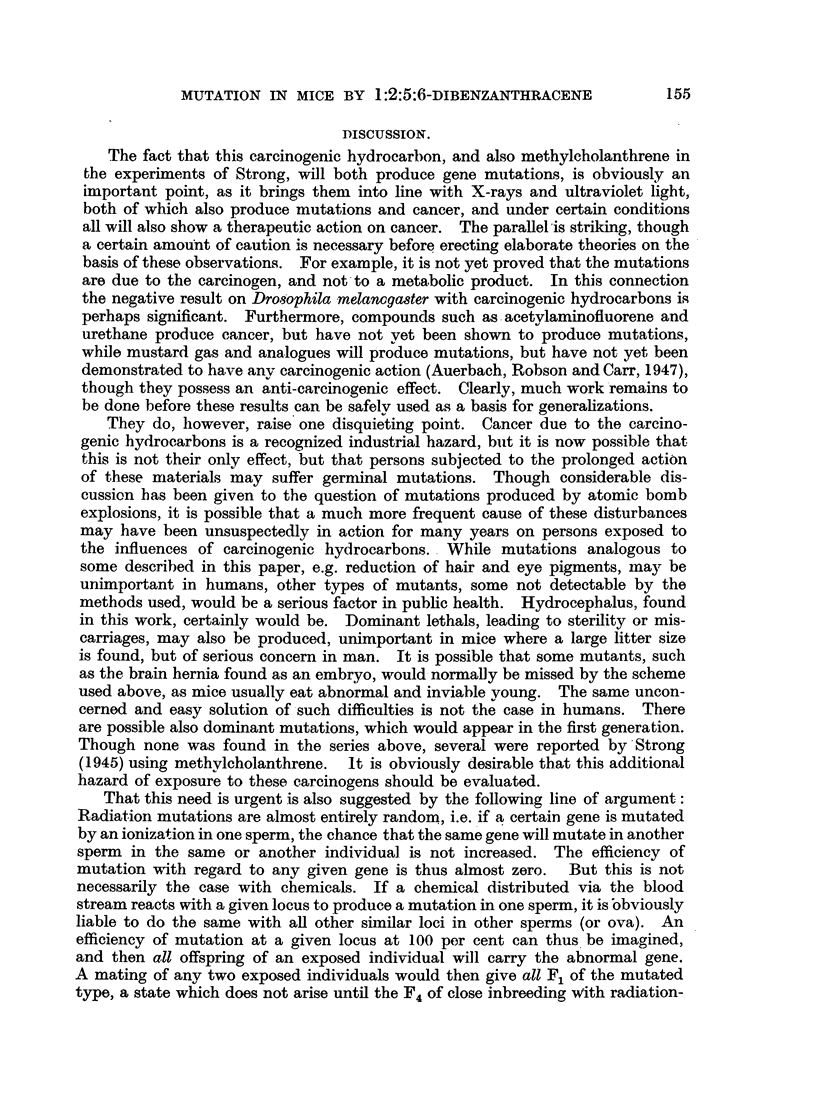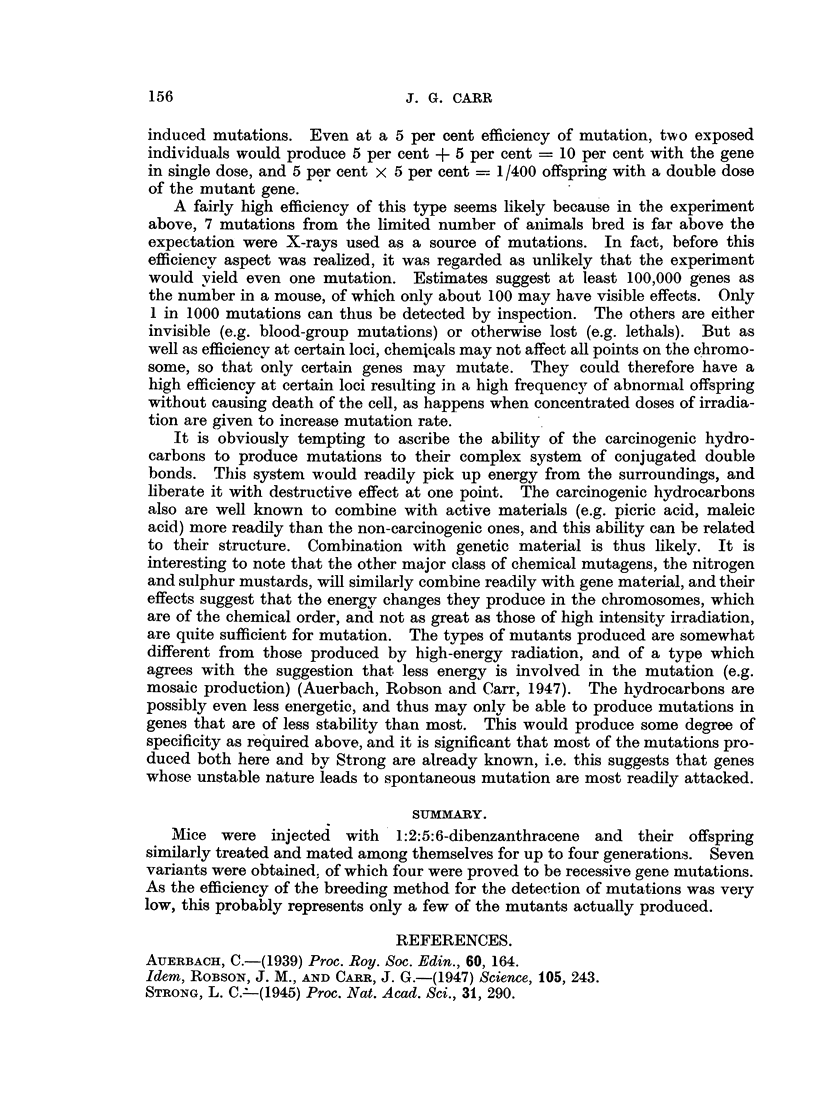# Production of Mutations in Mice by 1:2:5:6-Dibezanthracene

**DOI:** 10.1038/bjc.1947.17

**Published:** 1947-06

**Authors:** J. G. Carr


### PRODUCTION OF MUTATIONS IN MICE BY 1:2:5:6-DIBENZANTHRACENE.

J. G. CARR.

*From Institute of Animal Genetics, Edinburgh.*

Received for publication April 10, 1947.

IN an experiment in which mice of lines CBA, JK and N were crossed, and the offspring injected with methylcholanthrene for many generations, Strong (1945) made the important discovery that genetic mutations arose as a result of the injection of the carcinogenic compound. This substance had previously been tested on *Drosophila melanogaster* (Auerbach, 1939), and found not to produce lethal mutations in this species. In an experiment in which 1:2:5:6-dibenzanthracene was being given to mice, and the offspring used for breeding, it was regarded as possible that mutations might appear, though because of the nature and size of the experiment it was felt that the chance of any being detected was so small as to be negligible. Actually several mutations were obtained.

### METHOD.

If a mutation is produced in a gene of the sperm or ovum of a mouse, the offspring to which this gamete gives rise will be heterozygous for the mutation, and one-half of the sperm or ova it produces will carry the mutation. Since mutations are usually recessive they will only be detected by an inbreeding programme carried out for three generations after the mutation arises. This condition was partially fulfilled in the experiment to be described, which was actually set up with an entirely different aim.

*Materials.*—The experiment was part of a study of the effect of the dominant yellow ($A^Y$) gene on cancer susceptibility. Three inbred lines of mice were used, $C_3H$, CBA and RIII.

The first two lines are essentially wild-type mice, while the RIII is similar except for the presence of albinism. No phenotypically detectable mutations have arisen in any of these lines among thousands raised at this Institute, so frequent spontaneous mutations can be ruled out as a source of error. The $A^Y$ gene is a dominant giving a beautiful yellow coloration to the coat of the mice carrying it, and is not known to exist in the pure ($A^YA^Y$) state. All yellow mice thus carry a lower allelomorph of the $A$ series, and pure-breeding yellows are unknown. The $A^Y$ gene in the present experiment was derived from a stock kept by Dr. Auerbach, in which it is combined with the $a^t$ gene. This stock is inbred, though not by strict brother-sister matings. It would be impossible that all the mutations to be described could have come from this source, firstly because none has appeared in the $A^Ya^t$ stock despite inbreeding, and secondly because if they were so introduced, they should have appeared frequently in the *early*

generations of the experiment, while the mutations appeared, as would be expected, seldom and in the $F_3$ or later generations. A single male introduced the $A^Y$ gene into all three lines, and the yellow mice in the offspring were crossed again to the respective pure line, and this process carried on indefinitely. The end result will thus be mice essentially of pure-line make-up, but half of each litter will carry the $A^Y$ gene. Thus the susceptibility of mice genetically uniform except for presence of absence of the $A^Y$ gene can be studied.

As it was also felt desirable to study the effect of carcinogens on the $A^Y$ gene in non-uniform genotypes, some yellow mice of the $F_1$ and $F_2$ backcross (i.e. 1/2 or 3/4 pure-line, 1/2 or 1/4 $A^Y$ stock) were injected with carcinogen ($P_1$ treated generation), mated together, and the yellow offspring ($F_1$ treated) selected, injected with carcinogen and mated amongst themselves again. This was done for one more generation ($F_2$ treated), and partially for another generation ($F_3$ treated). A few $F_4$ mice were also raised. The carcinogen used was 1:2:5:6-dibenzanthracene, of which 0·1 c.c. of a saturated solution in arachis oil was injected subcutaneously at the age of 5–8 weeks. In all, 83 mice were treated. As the aim of the experiment was to study the histological type of tumour produced, litters were not kept and mated separately, but usually 2, 3, or 4 litters were mixed and allowed to breed together. This out-breeding must have seriously reduced the possibility of detecting mutations, and made it impossible to detect in which mouse the mutation arose. Detailed breeding records are thus not available, though it can be stated that all mutations appeared in the $F_3$ or later, and none in the $F_1$, which would be the case if the original yellow mouse was heterozygous for any mutation (see below).

It should be pointed out that the efficiency of this breeding arrangement for the detection of mutations was further reduced by three additional factors. Firstly, when yellow mice are bred together, all $A^YA^Y$ mice, which comprise one quarter of the offspring, die before birth, and any mutations which were associated with these individuals are lost. This reduces the efficiency to 75 per cent of the possible. Secondly, about 20 per cent of the offspring were albinos, resulting from the albino genes of the RIII yellows coming together, and colour variations are invisible in such mice. This reduced the efficiency by a further 20 per cent, so that the detection of mutants was only 60 per cent of the possible efficiency. Thirdly, many mutants visible on an agouti background (e.g. brown) are not shown when the yellow gene is present. This further reduced the efficiency.

*Mutants.*—As described above, all mice born in this experiment should have been of three types : normal agouti, albino, or yellow. The following variants were found in addition, in the $F_3$ treated and later generations :—

1. Hydrocephalus : Two mice, both albinos, with typical hydrocephalus arose in different litters in one cage. Both died at the age of 14 days, and the parents were not traced. It is possible that they were the two mice which died of cancer resulting from the injection of the hydrocarbon shortly after the second litter was produced. Breeding tests were thus impossible, but it should be noted that hydrocephalus is a well-known genetic mutation in mice. It has never been seen in inbred mice or in the $A^Y$ line mice at the Institute.

2. Absence of left horn of uterus : Found in one yellow mouse post-mortem. The parents were dead, and the genetic nature of this variant could not, therefore, be established. Nothing like this has ever been noted in the Institute mice.

3. Brain hernia : Found in one of seven 16-day embryos in a tumour-bearing yellow female. Nothing of this type has been noted in over 600 embryos of inbred lines examined, though again the genetic nature cannot be established.

4. " Brown " : Resembles the common recessive mutation *b*, and is inherited as a simple recessive.

5. " Pink eye " : Resembles the recessive genes *p* and *pa*, and is inherited as a simple recessive. Another appearance of a similar mutant, almost certainly independent, was lost before testing could be carried out.

6. " Recessive spotting " : Inherited as a recessive, and resembles the gene *s*.

7. Chinchilla : Resembles the recessive gene $c^{Ch}$, and like it is inherited as a recessive, allelomorphic with albinism (*c*). This mutation segregated twice during the breeding. It is possible from the breeding records that it arose from the same $P_1$ male (one of three in the same cage). If it arose from two mutated sperms it will represent two separate mutations. If the mutation arose in the earlier series of the germ-tract cells, it is possible that more than one sperm carrying the mutation was produced by this male, though the likelihood of these few out of the millions of sperm available being the ones to fertilize twice are remote. This second mutation is therefore probably independent, but this cannot be certain.

No attempt was made to breed further to obtain more mutants, though this might have been possible, as it was felt that a new and more efficient breeding method would give better results with the same labour.

*Contamination.*—In all mutation experiments the question of contamination, as opposed to mutation, arises. There are three possibilities in the present experiment, which, as will be shown, can be reasonably eliminated :—

(1) Mutants introduced by the inbred lines : As thousands of these have been raised at the Institute, all by strict brother-sister mating, without any mutants being found, it is extremely unlikely that these mutants could have been introduced by the inbred lines without detection. Furthermore, were this the case, some of the $F_1$ and $F_2$ of the mating with the $A^Y$ mouse (which were the $P_1$ treated generation) would have been heterozygous for these genes, and the mutations would have appeared in quantity in the next generation of crossing, i.e. the $F_1$ treated. This was not the case.

(2) For the same reasons the mutations could not have been present in the original $A^Y$ male, or they would have appeared in the yellow line or $F_1$ treated generation. Were he responsible, he would have had to be heterozygous for these 7 genes in addition to his own $A^Ya^t$ make-up, which would be rather an admirable genetic achievement, even if deliberately attempted.

(3) Contamination by stray mice : The whole experiment was carried out in a small room of the mousery, in which only this " yellow experiment " and inbred mice are maintained. Most of the rest of the cages were used for inbreeding experiments, which would detect any contamination by strays. No unexpected colours have ever occurred in the inbred mouse section, comprising nearly 500 breeding pairs. Contamination is thus unknown in a large control series. Furthermore, the " general purpose" mice in the mousery are of a much heavier type than the rather weedy inbreds, and on crossing with inbreds produce a strikingly larger mouse. None of the mutants was of this type, but all of the usual " inbred " size. Contamination by strays does not therefore appear to be a satisfactory explanation, and true genetic mutation must have occurred.

### DISCUSSION.

The fact that this carcinogenic hydrocarbon, and also methylcholanthrene in the experiments of Strong, will both produce gene mutations, is obviously an important point, as it brings them into line with X-rays and ultraviolet light, both of which also produce mutations and cancer, and under certain conditions all will also show a therapeutic action on cancer. The parallel is striking, though a certain amount of caution is necessary before erecting elaborate theories on the basis of these observations. For example, it is not yet proved that the mutations are due to the carcinogen, and not to a metabolic product. In this connection the negative result on *Drosophila melanogaster* with carcinogenic hydrocarbons is perhaps significant. Furthermore, compounds such as acetylaminofluorene and urethane produce cancer, but have not yet been shown to produce mutations, while mustard gas and analogues will produce mutations, but have not yet been demonstrated to have any carcinogenic action (Auerbach, Robson and Carr, 1947), though they possess an anti-carcinogenic effect. Clearly, much work remains to be done before these results can be safely used as a basis for generalizations.

They do, however, raise one disquieting point. Cancer due to the carcinogenic hydrocarbons is a recognized industrial hazard, but it is now possible that this is not their only effect, but that persons subjected to the prolonged action of these materials may suffer germinal mutations. Though considerable discussion has been given to the question of mutations produced by atomic bomb explosions, it is possible that a much more frequent cause of these disturbances may have been unsuspectedly in action for many years on persons exposed to the influences of carcinogenic hydrocarbons. While mutations analogous to some described in this paper, e.g. reduction of hair and eye pigments, may be unimportant in humans, other types of mutants, some not detectable by the methods used, would be a serious factor in public health. Hydrocephalus, found in this work, certainly would be. Dominant lethals, leading to sterility or miscarriages, may also be produced, unimportant in mice where a large litter size is found, but of serious concern in man. It is possible that some mutants, such as the brain hernia found as an embryo, would normally be missed by the scheme used above, as mice usually eat abnormal and inviable young. The same unconcerned and easy solution of such difficulties is not the case in humans. There are possible also dominant mutations, which would appear in the first generation. Though none was found in the series above, several were reported by Strong (1945) using methylcholanthrene. It is obviously desirable that this additional hazard of exposure to these carcinogens should be evaluated.

That this need is urgent is also suggested by the following line of argument: Radiation mutations are almost entirely random, i.e. if a certain gene is mutated by an ionization in one sperm, the chance that the same gene will mutate in another sperm in the same or another individual is not increased. The efficiency of mutation with regard to any given gene is thus almost zero. But this is not necessarily the case with chemicals. If a chemical distributed via the blood stream reacts with a given locus to produce a mutation in one sperm, it is obviously liable to do the same with all other similar loci in other sperms (or ova). An efficiency of mutation at a given locus at 100 per cent can thus be imagined, and then *all* offspring of an exposed individual will carry the abnormal gene. A mating of any two exposed individuals would then give *all* $F_1$ of the mutated type, a state which does not arise until the $F_4$ of close inbreeding with radiationinduced mutations. Even at a 5 per cent efficiency of mutation, two exposed individuals would produce 5 per cent + 5 per cent = 10 per cent with the gene in single dose, and 5 per cent × 5 per cent = 1/400 offspring with a double dose of the mutant gene.

A fairly high efficiency of this type seems likely because in the experiment above, 7 mutations from the limited number of animals bred is far above the expectation were X-rays used as a source of mutations. In fact, before this efficiency aspect was realized, it was regarded as unlikely that the experiment would yield even one mutation. Estimates suggest at least 100,000 genes as the number in a mouse, of which only about 100 may have visible effects. Only 1 in 1000 mutations can thus be detected by inspection. The others are either invisible (e.g. blood-group mutations) or otherwise lost (e.g. lethals). But as well as efficiency at certain loci, chemicals may not affect all points on the chromosome, so that only certain genes may mutate. They could therefore have a high efficiency at certain loci resulting in a high frequency of abnormal offspring without causing death of the cell, as happens when concentrated doses of irradiation are given to increase mutation rate.

It is obviously tempting to ascribe the ability of the carcinogenic hydrocarbons to produce mutations to their complex system of conjugated double bonds. This system would readily pick up energy from the surroundings, and liberate it with destructive effect at one point. The carcinogenic hydrocarbons also are well known to combine with active materials (e.g. picric acid, maleic acid) more readily than the non-carcinogenic ones, and this ability can be related to their structure. Combination with genetic material is thus likely. It is interesting to note that the other major class of chemical mutagens, the nitrogen and sulphur mustards, will similarly combine readily with gene material, and their effects suggest that the energy changes they produce in the chromosomes, which are of the chemical order, and not as great as those of high intensity irradiation, are quite sufficient for mutation. The types of mutants produced are somewhat different from those produced by high-energy radiation, and of a type which agrees with the suggestion that less energy is involved in the mutation (e.g. mosaic production) (Auerbach, Robson and Carr, 1947). The hydrocarbons are possibly even less energetic, and thus may only be able to produce mutations in genes that are of less stability than most. This would produce some degree of specificity as required above, and it is significant that most of the mutations produced both here and by Strong are already known, i.e. this suggests that genes whose unstable nature leads to spontaneous mutation are most readily attacked.

### SUMMARY.

Mice were injected with 1:2:5:6-dibenzanthracene and their offspring similarly treated and mated among themselves for up to four generations. Seven variants were obtained, of which four were proved to be recessive gene mutations. As the efficiency of the breeding method for the detection of mutations was very low, this probably represents only a few of the mutants actually produced.

### REFERENCES.

Auerbach, C.—(1939) *Proc. Roy. Soc. Edin.*, **60**, 164.
*Idem*, Robson, J. M., and Carr, J. G.—(1947) *Science*, **105**, 243.
Strong, L. C.—(1945) *Proc. Nat. Acad. Sci.*, **31**, 290.